# Supplementary material for: Effects of unilateral posterior missing-teeth on the temporomandibular joint and the alignment of cervical atlas
Source: PLoS One. 2020 Dec 2;15(12):e0242717. doi: 10.1371/journal.pone.0242717 (PMC7710100; doi:10.1371/journal.pone.0242717)
Supplement: Attachment — Submitted filename: Response to reviewer(plos one).docx [file pone.0242717.s003.docx]

**Manuscript ID:** PONE-D-20-23912
**Article Type:** Original article
**Title:** Effects of unipartite posterior missing-teeth on the temporomandibular joint and the alignment of cervical atlas.

**Dear Editor**:

We sincerely thanks you for giving us a chance to revise our manuscript. The reviewer's comments are constructive and valuable for improving our research. We have carefully read all comments and tried our best to revise the manuscript as per reviewers' suggestions. We have revised our paper and re-submitted it again. We believe that this paper will be of interest to your journal's readership because it introduces a new cause of TMJ degeneration. This knowledge can help prevent permanent and painful degeneration of TMJ in patients. We hope our research results may meet with acceptance requirements.

Sincerely,

Kuo-Chou Chiu

School of Dentistry, National Defense Medical Center

No. 325, Section 2, Cheng-Kung Road, Nei-hu, Taiwan R.O.C.

Phone: 886-2-87923311-13823

Fax: 886-2-87927147

E-mail address: scalingdentist61@yahoo.com.tw

**Response to the reviewer’s comments**

**Reviewer #1**

**Question 1:**
 The term “Unipartite” used to describe unilateral missing molars undoes not appear in the literature (trending)and confuses the reader. As well it may accurately indicate a missing tooth but not whether there was missing posterior support unilaterally or not.

**Response:**

Thank you very much for your valuable suggestions. Your suggestions made a vast improvement in our research paper. We analyzed and compared patients with one-side missing one or more posterior teeth, the changing of articular eminence and cranio-cervical angle with patients without missing teeth, and trying to provide valuable clinical data on temporal mandibular joint research. We accepted your suggestion and corrected the word "unipartite" into "unilateral" in the revised version with yellow highlighted in the revised version. We hope this can improve its readability in our article.

**Question 2:**

Was cost or availability the only reason the radiographic use of a CBCT not employed in this study?

**Response:**

The cost availability approach, radiation dose, and cost-effectiveness were the main concerned while we designed the research. It is also the most straightforward approach for a clinician to apply in their daily works. The CBCT delivered more than five to sixteen times the radiation dose than the panoramic film.^1^ Although CBCT may provide a more detailed image with anatomy landmarks or pathological lesions, the radiation dose is the primary concern due to radiation associated with cancer development.^2^ The field of view (FOD) with CBCT is another consideration. The FOV of dental CBCT is around 16X13 cm. This FOV may not cover all cervical atlas and TMJ in one field. Conventional CT may provide better FOD, but with higher radiation exposure. We wish to share our findings with cheaper, low-dose risk, and accessible approach radiology method at the beginning. In the future, we will try to conquer the limitation of CBCT and provide more information to the public.

**Question 3:**

Was there a rational for the sample size provided?

**Response:**

Although the sample size is not big, we have double confirmed that our experimental design is rational by two biostatisticians. It is not easy to collect samples both with panoramic film and cephalometric film in a dental clinic. We treasure the resources we have, and we will continue to follow this research in the future.

**Question 4:**

Was a lack of unilateral support in the posterior a criteria, or just a single missing tooth sufficient to be included in the study?

**Response:**

The criteria we included were lack of unilateral support posterior tooth, a single or more than one missing posterior molars. Our unpublished data found an interesting trend that some young people missed their unilateral posterior teeth for a time and then have temporal mandibular problems, but most edentulous elderly seldom complained of temporal mandibular disease. That is why we start to focus on this issue. We have modified our revised version with highlights changes in our revised version to reduce the confusion in material and method.

**Question 5:**

As the age range is large (20-69 years of age) and "average" age listed (36) may not well represent the possible changes, these participants may have the difference of 49 years of life.

**Response:**

We appreciate the reviewer's suggestion. That is also what we are concerned about since our sample is not a big database. However, our hospital is a military hospital. There is more younger population in our clinic. The standard deviation is complete (12.83). However, our results echoed with our clinical findings that it seemed to tend that younger people with unilateral missing posterior teeth may have more temporal-mandibular problems. We will continue to collect more data to confirm our findings.

**Question 6:**

Perhaps a listing of the average age of both arms of the study (missing teeth/ non-missing teeth) could be included?

**Response:**

Thanks for your valuable suggestion. In this study, the samples were randomized selected and collected but were not equal to stratified samples selected. Besides, the sample size is not very big, although it is rational proved. Listing of both arms' average age in this study may be not objective because it is not an equal stratified sample selected and collected.

Reference

1. Robers J, Drage N, Davies J, Thomas D. Effective dose from cone beam CT examinations in dentistry. Br J Radiol. 2009;82(973):35-40
2. Lorenoni DC, Bolognese AM, Garn DG, Gudedes FR, Sant’Anna EF. Conebeam computed tomography and radiograph in dentistry: aspects related to radiation dose. Int J Dent. 2012;2012:813768. Heeps://doi.org/10.1155/2012/813768
3. Henry E, Gansky SA, Lee JS, Johnson E, Maki K, Miller AJ, Hang JC. Comparative analysis of traditional radiographs and con-beam computed tomography volumetric images in the diagnosis and treatment planning of maxillary impacted canines. Am. J Orthod Dentofac Orthop. 2010; 137(5):590-7
