# Supplementary material for: Effects of unilateral posterior missing-teeth on the temporomandibular joint and the alignment of cervical atlas
Source: PLoS One. 2020 Dec 2;15(12):e0242717. doi: 10.1371/journal.pone.0242717 (PMC7710100; doi:10.1371/journal.pone.0242717)
Supplement: Attachment — Submitted filename: Reviewer Comments for PONE-D-20-23912.pdf [file pone.0242717.s002.pdf]

PONE-D-20-23912

Effects of unipartite posterior missing-teeth on the temporomandibular joint and the alignment of cervical atlas

Tsun-Hung Fang , Meng-Ta Chiang , Ming-Chun Hsieh , Ling-Yu Kung , Kuo-Chou Chiu

<u>Title</u>
The term “Unipartite” used to describe unilateral missing molars undoes not appear in the literature (trending) and confuses the reader. As well it may accurately indicate a missing tooth but not whether there was missing posterior support unilaterally or not.

<u>Introduction</u>
Authors have noted their previous study looking at the angle of condylar guidance and suggested the use of a Cone Beam Cat Scan (CBCT) for future studies.

Was cost or availability the only reason the radiographic use of a CBCT not employed in this study?

Methods
Was there a rational for the sample size provided?

Was a lack of unilateral support in the posterior a criteria, or just a single missing tooth sufficient to be included in the study?

Results
As the age range is large (20 - 69 years of age) and ‘average’ age listed ((36) may not well represent the possible changes these participants may have with the difference of 49 years of life.

Perhaps a listing of average age of both arms of the study (missing teeth/ non-missing teeth) could be included?

I am not able to speak to the statistical significance results. It appears that the figures accurately represent the data.

Discussion

Good discussion with limitations of study and future studies to be done. I found the discussion accurate and well written with good conclusions.

I feel that this is a good and valid study
